# Orange Fluorescent Proteins: Structural Studies of LSSmOrange, PSmOrange and PSmOrange2

**DOI:** 10.1371/journal.pone.0099136

**Published:** 2014-06-24

**Authors:** Sergei Pletnev, Daria M. Shcherbakova, Oksana M. Subach, Nadya V. Pletneva, Vladimir N. Malashkevich, Steven C. Almo, Zbigniew Dauter, Vladislav V. Verkhusha

**Affiliations:** 1 Leidos Biomedical Research Inc., Basic Research Program, Argonne, Illinois, United States of America; 2 Macromolecular Crystallography Laboratory, National Cancer Institute, Argonne, Illinois, United States of America; 3 Department of Anatomy and Structural Biology, Albert Einstein College of Medicine, Bronx, New York, United States of America; 4 Department of Biochemistry, Albert Einstein College of Medicine, Bronx, New York, United States of America; 5 Shemyakin–Ovchinnikov Institute of Bioorganic Chemistry, Russian Academy of Sciences, Moscow, Russian Federation; 6 Department of Nano, Bio, Information and Cognitive Technologies, Moscow Institute of Physics and Technology, Moscow, Russian Federation; Molecular Biology Institute of Barcelona, CSIC, Spain

## Abstract

A structural analysis of the recently developed orange fluorescent proteins with novel phenotypes, LSSmOrange (λ_ex_/λ_em_ at 437/572 nm), PSmOrange (λ_ex_/λ_em_ at 548/565 nm and for photoconverted form at 636/662 nm) and PSmOrange2 (λ_ex_/λ_em_ at 546/561 nm and for photoconverted form at 619/651 nm), is presented. The obtained crystallographic structures provide an understanding of how the ensemble of a few key mutations enabled special properties of the orange FPs. While only a single Ile161Asp mutation, enabling excited state proton transfer, is critical for LSSmOrange, other substitutions provide refinement of its special properties and an exceptional 120 nm large Stokes shift. Similarly, a single Gln64Leu mutation was sufficient to cause structural changes resulting in photoswitchability of PSmOrange, and only one additional substitution (Phe65Ile), yielding PSmOrange2, was enough to greatly decrease the energy of photoconversion and increase its efficiency of photoswitching. Fluorescence of photoconverted PSmOrange and PSmOrange2 demonstrated an unexpected bathochromic shift relative to the fluorescence of classic red FPs, such as DsRed, eqFP578 and zFP574. The structural changes associated with this fluorescence shift are of considerable value for the design of advanced far-red FPs. For this reason the chromophore transformations accompanying photoconversion of the orange FPs are discussed.

## Introduction

Fluorescent proteins (FPs) of the GFP-like family have become valuable tools for molecular biology, biochemistry, and biomedicine. Most challenging task of the FPs studies was the development of FPs with longer excitation/emission wavelength. This pursuit was motivated by advantages of red-shifted FPs, namely, lower background of cellular autofluorescence in microscopy, lower light scattering, and reduced tissue absorbance of longer wavelengths for *in vivo* imaging. In addition to common FPs, there are proteins of other phenotypes available, including FPs with a large Stokes shift (LSS) and irreversibly and reversibly photoswitchable (PS) FPs [1].

According to their emission wavelength, red-shifted FPs could be divided in the following groups: 520–540 nm yellow FPs (YFPs), 540–570 nm orange FPs (OFPs), 570–620 nm red FPs (RFPs), and >620 nm far-red FPs. Red shift of fluorescence of these FPs is predominantly achieved by extension of the conjugated system of the chromophore and its protonation/deprotonation. The variety of spectral properties of FPs, such as excitation and emission wavelength, quantum yield, brightness, photoswitchability, Stokes shift of fluorescence, result from different chromophore structures and its interactions with surrounding amino acid residues.

**Figure 1:**
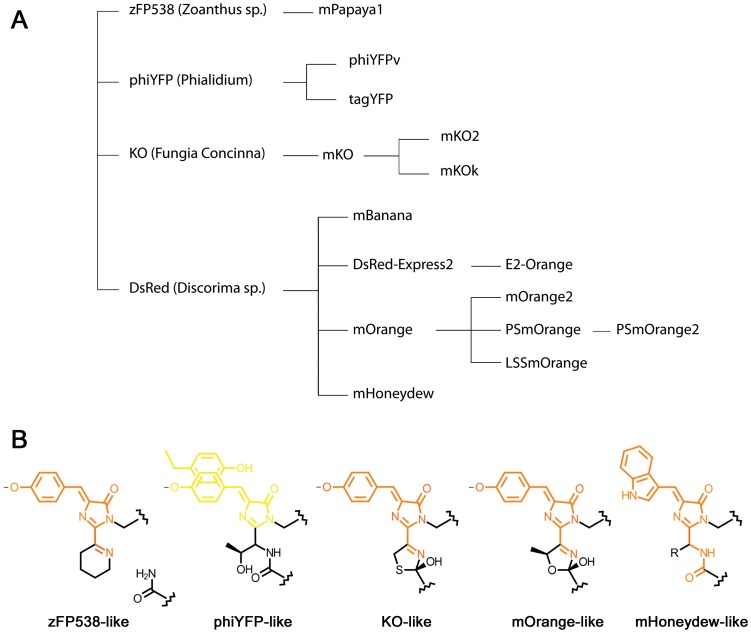
Evolution of the subfamily of orange fluorescent proteins. (**A**) Phylogenic tree showing the history of the development of different orange fluorescent proteins. (**B**) Chemical structures of the chromophores found in orange fluorescent proteins.

OFPs fill up a spectral gap between YFPs and RFPs and enable four-color imaging together with blue, green, and far-red FPs. The spectral properties of OFPs mainly result from their chromophore structures. The chromophores of OFPs are formed by conservative -X-Y-G- tri-peptides in which X =  Thr, Lys, Ser or Cys. In most cases, their orange emission is attributed to a three-ring chromophore structure with the third cycle formed by cyclization of the side chain of the X residue, extending the π-electron system of the chromophore [2]. Recombinant OFPs, reported so far, have been engineered from four wild-type proteins: zFP538 (*Zoanthus sp*.) [3], KO *(Fungia concinna)* [4], DsRed *(Discosoma sp.)* [5] and phiYFP *(Phialidium)* [6] (Figure 1, Table 1).

**Table 1:** Spectral and evolutionary features of orange fluorescent proteins.

Fluorescent protein	λ_ex_^max^/λ_em_^max^	E_mol_	Φ_F_ 3	B 4	B_rel_^EGFP^ 5	Reference
	(nm) 1	(M^−1^ cm^−1^) 2				
EGFP *	489/509	53,000	0.6	31.8	1	[52]
*(Zoanthus sp.)*						
zFP538	528/538	20,200	0.42	8.5	0.27	[3]
mPapaya1	530/541	43,000	0.83	35.7	1.12	[10]
*(Phialidium)*						
phiYFP	525/537	115,000	0.6	69	2.17	[6]
phiYFPv	524/537	101,305	0.59	59.8	1.88	[11]
TagYFP	508/524	20,056	0.74	14.8	0.47	[11]
*(Fungia concinna)*						
KO	548/561	109,750	0.45	49.4	1.55	[4]
mKO	548/559	51,600	0.6	31	0.97	[4]
mKO2	551/565	63,800	0.57	36.4	1.14	[13]
mKOκ	551/563	105,000	0.61	64.1	2.01	[14]
*(Discosoma sp.)*						
DsRed**	558/583	75,000	0.7	52.5	1.65	[5]
mHoneydew	504/562	17,000	0.12	2	0.06	[2]
mBanana	540/553	6,000	0.7	4.2	0.13	[2]
mOrange	548/562	71,000	0.69	49	1.54	[2]
mOrange2	549/565	58,000	0.6	34.8	1.09	[15]
LSSmOrange	437/572	52,000	0.45	23.4	0.74	[19]
PSmOrange	548/565	113,300	0.51	57.8	1.82	[20]
PSmOrange2	546/561	51,000	0.61	31.1	0.98	[21]
DsRed-Express2**	554/591	35,600	0.42	15	0.47	[16]
E2-Orange	540/561	36,500	0.54	19.7	0.62	[18]

*The values for EGFP are given for comparison.

**DsRed and DsRed-Express2 are red fluorescent proteins.

1 λ_ex_^max^/λ_em_^max^ - excitation and emission maxima.

2 E_mol_ - extinction coefficient.

3 Φ_F_ -quantum yield.

4 B - brightness (E_mol_ × Φ_F_)/1000.

5 B_rel_^EGFP^ - brightness relative to EGFP (product of Φ_F_ and E_mol_ compared to the brightness of EGFP (53,000 M^−1^ cm^−1^×0.60) [52]).

zFP538 is an obligate tetramer of low brightness with -K-Y-G- chromophore triad. Cyclization of Lys is accompanied by a cleavage of the polypeptide chain between the chromophore and the preceding amino acid residue, F65 [7]–[9]. Multiple rounds of directed evolution of zFP538 yielded its bright monomeric version, mPapaya1 [10], useful both as a protein tag and as a donor in orange-red Förster resonance energy transfer (FRET) pairs. The chromophore of phiYFP consists of -T-Y-G- residues. Its crystal structure shows that the side chain of Thr does not form a third cycle present in zFP538 and its yellow-orange emission was attributed to the antiparallel stacking between Y203 and the chromophore [11]. Two monomeric variants of phiYFP with improved folding, preserving this structural feature, phiYFPv and TagYFP, have been reported and described in [11]. The chromophore of KO is formed by -C-Y-G- amino acids and cyclization of Cys results in the third cycle, extending the chromophore conjugation system [12]. However, unlike in zFP538, the cyclization does not result in the cleavage of the polypeptide backbone. The monomeric form of KO with an improved folding, mKO, has been reported and characterized [4]. mKO has been further used to generate two variants with the improved brightness, mKO2 [13] and mKOk [14]. Most prolific FP, which yielded a total of eight OFPs, is DsRed [5]. Red emission of its -Q-Y-G- chromophore was attributed to the presence of acylimine group connecting the imidazolinone ring of the chromophore with the preceding amino acid residue (F65) extending its conjugation system [5]. Variation of the DsRed chromophore tripeptide composition resulted in three OFPs: mHoneydew (-M-W-G-), mBanana (-C-Y-G-) and mOrange (-T-Y-G-) [2]. mOrange was shown to be the brightest and was used for generation of its more photostable variant mOrange2 [15]. Additionally, DsRed was used to obtain an RFP with low cytotoxicity, DsRed-Express2 [16], [17], that served as a template for generation of its low cytotoxic orange variant E2-Orange [18].

Recently, three OFPs with novel phenotypes have been developed: permanently fluorescent LSSmOrange with a large Stokes shift (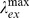
437 nm, 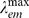 572 nm) [19] and two proteins photoswitchable from orange to far-red fluorescent states PSmOrange (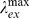 548 nm, 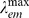
565 nm; photoconverted form 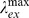 636 nm, 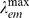
662 nm) [20] and PSmOrange2 (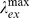 546 nm, 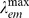 561 nm; photoconverted form 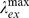 619 nm, 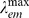
651 nm) [21].

LSSmOrange fills a spectral gap between green-yellow and red LSSFPs. Its brightness is five-fold larger than that of the brightest red LSSFP and is similar to that of green-yellow LSSFPs. LSSmOrange was shown to be useful for multicolor imaging, simultaneous detection of two Förster resonance energy transfer (FRET)-based biosensors using a single excitation wavelength, and single-laser fluorescence cross-correlation spectroscopy (FCCS) imaging of four FPs [19]. PSmOrange could be used for multicolor photolabeling, super-resolution photoactivated localization microscopy (PALM), and tracking of the photoswitched cell population in living animals [20]. PSmOrange2 has been shown to exhibit a higher rate and improved efficiency of photoconversion and enable a unique phenomenon, termed FRET-facilitated photoswitching [21].

Here, we report the X-ray crystal structures of LSSmOrange, PSmOrange, and PSmOrange2, focus on peculiarities of the chromophores and their immediate environments, and discuss the mechanistic basis of the unique LSS and PS properties of FPs.

## Materials and Methods

### Protein Expression and Purification

For crystallization, PCR-amplified *Bgl*II/*Eco*RI fragments encoding LSSmOrange, PSmOrange and PSmOrange2 were cloned into a pBAD/His-B vector (Invitrogen), modified by shortening the N-terminal polyhistidine tag to the MGSHHHHHHGRS- amino acid sequence. All proteins were expressed in LMG194 bacterial host (Invitrogen) grown in a minimal medium (RM) supplemented with 0.005% arabinose at 37°C for 24 h and at 25°C for 24 h upon shaking at 200 rpm. The cells were pelleted down by centrifugation, re-suspended in phosphate buffer saline, and lysed by sonication. The proteins were purified using Ni-NTA agarose (Qiagen) and size exclusion chromatography using Superdex 200 (16/60) column (GE Healthcare) followed by the dialysis against 10 mM NaH_2_PO_4_, pH 7.5.

### Crystallization

For crystallization, LSSmOrange was transferred to a buffer containing 10 mM Tris-HCl, 100 mM NaCl, 2.5 mM EDTA pH 8.0 and were concentrated to 25 mg/ml. An initial search for crystallization conditions was carried out using a Mosquito Robotic Crystallization System (TTP LabTech Ltd). Large-scale crystallization was set up by the hanging drop vapor diffusion method at room temperature. The best crystals for LSSmOrange were obtained from Wizard I crystallization kit (Emerald BioSystems), condition 48 (0.2 M Zn acetate, 0.1 M acetate buffer pH 4.5, 20% PEG 1000). Suitable for data acquisition crystals of PSmOrange and PSmOrange2 were obtained using the ComPAS crystallization suite (Qiagen), conditions C4 (55% MPD) and B8 (0.1 M HEPES, pH 7.5, 20% PEG 10000), respectively, in vapor diffusion sitting drop configuration.

### Data Acquisition

For LSSmOrange, crystals X-ray diffraction data were collected at the Advanced Photon Source on SER-CAT 22-ID beamline (Argonne National Laboratory). Diffraction intensities were registered on a MAR 300 CCD detector (Rayonix). Prior to data collection the crystals were incubated in a cryoprotecting solution consisting of 20% glycerol and 80% of well solution for 30 seconds and were flash-frozen in 100 K nitrogen stream. Cryogenic temperature was maintained by a CryoJetXL cooling device (Oxford Cryosystems). Diffraction images were indexed, integrated and scaled with the HKL2000 software [22].

Diffraction data of the PSmOrange and PSmOrange2 crystals were collected on a Quantum 315 CCD detector (Area Detector Systems) on the X29A beamline (National Synchrotron Light Source, Brookhaven National Laboratory). PSmOrange and PSmOrange2 crystals were mounted directly from the screening trays. Prior to freezing, 20% glycerol was added to PSmOrange2 crystals as cryoprotectant. Intensities were integrated using HKL2000 and reduced to amplitudes using TRUNCATE [23], [24]. Data processing statistics are given in Table 2.

**Table 2:** Data collection and refinement statistics.

Protein	PSmOrange	PSmOrange2	LSSmOrange
Space group	*P*1	*P*2_1_	*P*2_1_
Unit cell parameters			
a, b, c (Å)	45.4, 50.7, 52.4	45.7, 43.9, 52.5	37.4, 107.4, 56.6
α, β, γ (°)	94.7, 90.0, 106.6	90.0, 94.4, 90.0	90.0, 102.2, 90.0
Temperature (K)	100	100	100
Wavelength (Å)	1.08	1.08	1
Resolution (Å)	30.0–1.95	30.0–1.30	30.0–1.40
Total reflections	87,470	158,987	357,447
Unique reflections	26,506	46,761	84,546
Completeness^a^ (%)	84.6 (56.5)	96.3 (84.2)	98.6 (97.1)
I/σ(I)	9.9 (7.4)	12.2 (3.5)	24.8 (1.9)
R-merge^b^	0.055 (0.176)	0.050 (0.361)	0.056 (0.628)
Multiplicity	3.3 (2.9)	3.4 (2.2)	4.2 (4.1)
No. of protein atoms	3,513	1,929	3,812
No. of solvent atoms	161	212	301
Resolution range (Å)	30.0–1.95	30.0–1.30	30.0–1.40
R-work^c^	0.2	0.148	0.147
R-free^c^	0.266	0.177	0.175
R.m.s.d. bond lengths (Å)	0.018	0.014	0.014
R.m.s.d. angles (°)	2.15	1.59	1.98
R.m.s.d. chirality (Å^3^)	0.13	0.08	0.159
R.m.s.d. planarity (Å)	0.011	0.007	0.01
Ramachandran statistics (%)			
(for non-Gly/Pro residues)			
most favorable	92.7	93.9	95
additional allowed	7.3	6.1	5

a Data in parentheses are given for the outermost resolution shells: 1.98–1.95 Å for PSmOrange, 1.32–1.30 Å for PSmOrange2, and 1.45–1.40 Å for LSSmOrange.

b R_merge_  =  Σ_hkl_Σ_j_ |I_j_(hkl) – <I(hkl)>|/Σ_hkl_Σ_j_|<I(hkl)>|, where I_j_ is the intensity measurement for reflection j and <I> is the mean intensity over j reflections.

c R_work_/(R_free_)  =  Σ ||F_o_(hkl)| – |F_c_(hkl)||/Σ |F_o_(hkl)|, where F_o_ and F_c_ are observed and calculated structure factors, respectively. No σ-cutoff was applied. 5% of the reflections were excluded from refinement and used to calculate R_free_.

### Structure Solution and Refinement

The structure of LSSmOrange was solved by molecular replacement method with MOLREP [25] using as a search model a single monomer of mOrange (PDB ID: 2H5O, [8]), excluding its chromophore. Structure refinement was performed with REFMAC [26], [27] and COOT [28]. Manual structure rebuilding and addition of ordered solvent molecules were done using COOT. Structure validation was performed with COOT and PROCHECK [29].

The structures of PSmOrange and PSmOrange2 were determined by molecular replacement using PHASER [30]. Model building and refinement were performed with REFMAC and COOT. The quality of the final structure was verified with composite omit maps, and the stereochemistry was checked with MOLPROBITY [31]. The refinement statistics for LSSmOrange, PSmOrange and PSmOrange2 are given in Table 2.

## Results

**Figure 2:**
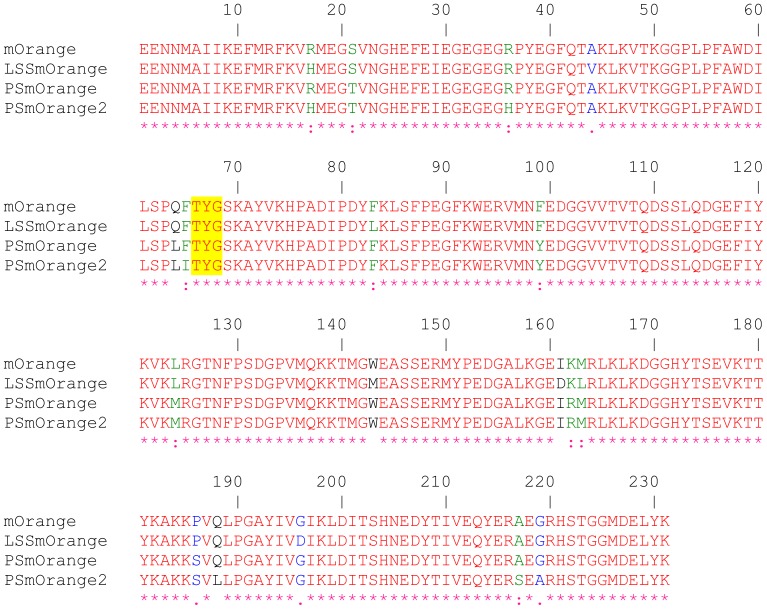
Amino acid alignment of mOrange, LSSmOrange, PSmOrange and PSmOrange2. The chromophore-forming tri-peptides are highlighted in yellow.

**Figure 3:**
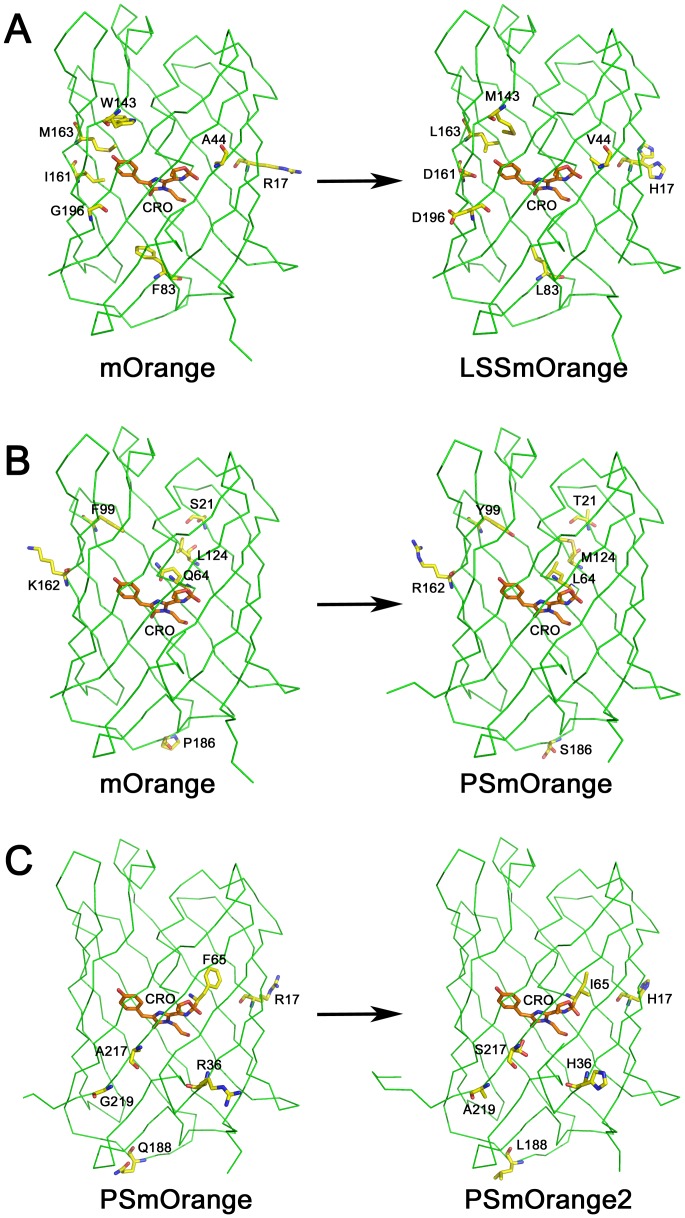
Amino acid differences between the parental and successor proteins in 3D. (**A**) The transformation of mOrange into LSSmOrange. (**B**) The transformation of mOrange into PSmOrange. (**C**) The transformation of PSmOrange into PSmOrange2.

LSSmOrange and PSmOrange have been engineered from mOrange and PSmOrange2 has been engineered from PSmOrange using directed molecular evolution. The resulted OFPs constitute the following mutants: mOrange/R17H/A44V/F83L/W143M/I161D/M163L/G196D (LSSmOrange), mOrange/S21T/Q64L/F99Y/L124M/K162R/P186S (PSmOrange), and PSmOrange/R17H/R36H/F65I/Q188L/A217S/G219A (PSmOrange2) (Figures 2, 3). Here and below the numbering is adopted from mOrange PDB file, 2H5O [8]. Note that not all of the listed mutations are located in the nearest chromophore environment. In fact, only three residues in LSSmOrange (M143, D161, and L163), one in PSmOrange (L64), and two in PSmOrange2 (I65 and S217) are situated close enough to the chromophore to directly affect its electronic properties (Figure 3).

### Crystal Structures of LSSmOrange, PSmOrange, and PSmOrange2

The asymmetric units of LSSmOrange and PSmOrange crystals contain two monomers oriented approximately parallel to each other, whereas the asymmetric unit of PSmOrange2 contains a single monomer. Crystallographic symmetry operations do not complete monomers to classic GFP-like tetramers indicating a true monomeric nature of the proteins. Multiple conformations are observed for the total of 30 (6.5%) amino acid residues of LSSmOrange and for 26 (11.3%) amino acid residues of PSmOrange2. Multiple conformations for PSmOrange were not modeled due to the relatively low (1.94 Å) resolution of the diffraction data.

The final models of LSSmOrange, PSmOrange, and PSmOrange2 have low deviations of bond length, angles, chiral volumes and planes from ideal values indicating its high quality. In all models, over 90% of the residues are located in the most favorable regions of the Ramachandran plot with the rest located in the additionally allowed regions (Table 2). Superposition of parental mOrange with LSSmOrange, PSmOrange and PSmOrange2 by Cα atoms resulted in root mean square deviations of 0.35 Å, 0.39 Å, and 0.38 Å, respectively, indicating close similarity of the structures.

### Chromophores of LSSmOrange, PSmOrange, and PSmOrange2 and their Environment

Electron density maps show that LSSmOrange, PSmOrange, and PSmOrange2 have the same chromophore structures as parental mOrange [8]. It consists of *p-*hydroxyphenyl, imidazolinone, and 2-hydroxy-dihydrooxazole rings. The latter one results from cyclization of T66 side chain. The chromophore adopts *cis-*conformations and in all examined FPs has a noticeable but not severe deviation from coplanarity between the adjacent *p-*hydroxyphenyl and imidazolinone rings (χ1, the rotation around Cα2-Cβ2 bond, is ∼5–10°). The largest deviation from coplanarity is observed between 2-hydroxy-dihydrooxazole and imidazolinone rings of the chromophore. The Cα1 atom of the chromophore has partially pyramidal geometry with the sum of the valent angles around it of ∼348°. Due to a distorted geometry of Cα1 atom, these rings are oriented at the angle of ∼30° with respect to each other. The deviation of Cα1 atom from planar sp^2^-geometry is presumably caused by a local steric strain between the chromophore and F65 (preceding amino acid residue).

**Figure 4:**
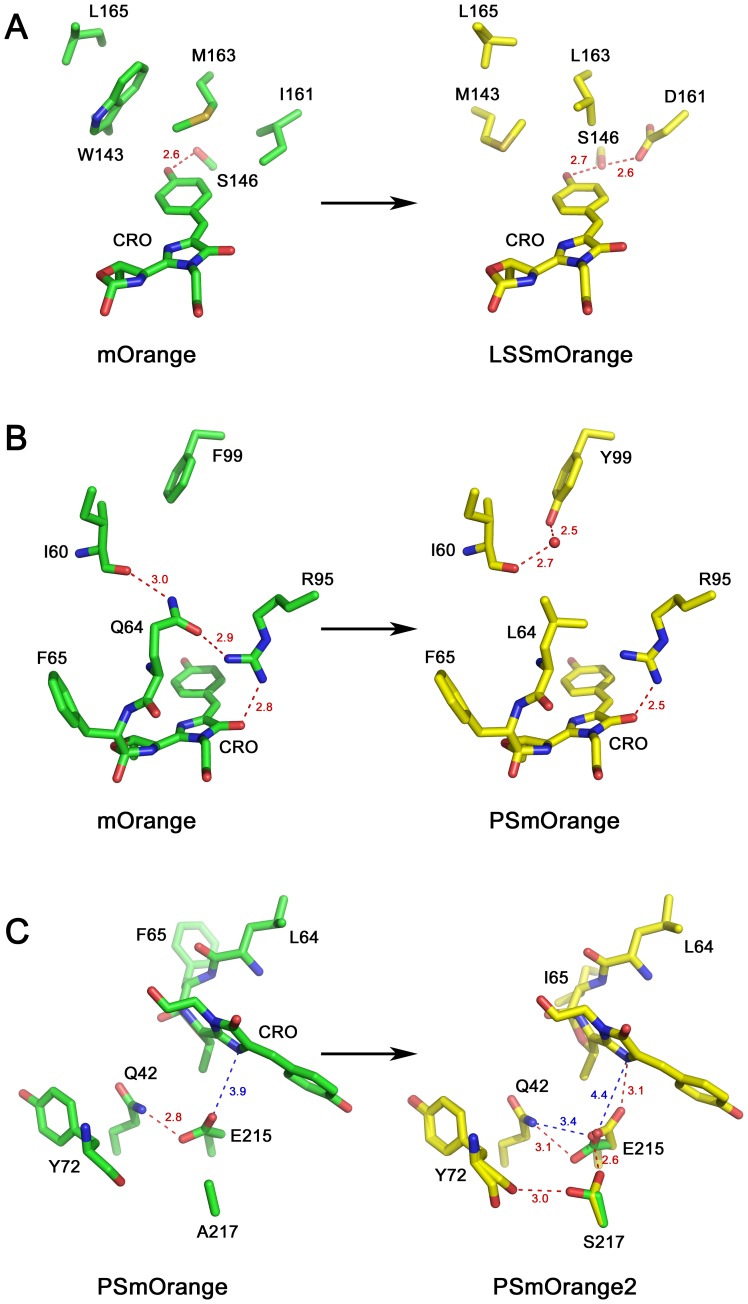
The differences in the immediate chromophore environment between the parental and successor proteins. (**A**) The difference between mOrange and LSSmOrange. (**B**) The difference between mOrange and PSmOrange. (**C**) The difference between PSmOrange and PSmOrange2.

Key substitutions in LSSmOrange responsible for its ∼135 nm large Stokes shift comprise I161D, M163L, and W143M enabling the excited state proton transfer (ESPT) [19]. These substitutions introduce noticeable changes in the nearest chromophore environment of LSSmOrange (Figure 4A). X-ray structure shows that W143M replacement provides a shift of the side chain of L165 towards protein interior, and both W143M and M163L substitutions improve the chromophore planarity relative to parental mOrange (Figure 4A). The χ1 angles of mOrange and LSSmOrange chromophores are 12.3° and 6.3°, respectively. In both mOrange and LSSmOrange, the oxygen atom of *p-*hydroxyphenyl group of the chromophore forms an H-bond with the side chain hydroxyl of S146. Rotation of the chromophore around Cα2-Cβ2 bond in LSSmOrange, arising from W143M and M163L mutations, causes the change of S146 side chain conformation. The hydroxyl atom of S146 now forms two H-bonds, one with the chromophore and another with the side chain of D161 (I161 in mOrange). As a result, S146 acts as a mediator for ESPT from *p-*hydroxyphenyl ring of the chromophore to carboxyl group of D161 (Figure 4A). Four other substitutions, R17H, A44V, F83L, and G196D, are located far from the chromophore and presumably improve folding efficiency, brightness and photostability (Figure 3A).

The key substitutions enabling PSmOrange photoconversion are Q64L and F99Y introduced in a process of molecular evolution via random and rational mutagenesis of parental mOrange [20]. In mOrange, Q64 forms H-bonds with guanidine group of R95 and the main chain carbonyl of I60 (Figure 4B). Substitution of Q64 with Leu disables formation of these two H-bonds (Figure 4B). The side chain carboxyl of Y99 forms new water-mediated H-bond with the main chain carbonyl of I60. These two changes result in the protein that in the presence of oxidizing agents such as potassium ferricyanide or intracellular oxidants and light could be converted into a far-red emitting form [20]. Out of six substitutions made in mOrange to obtain PSmOrange Q64L is the only one located close enough to the chromophore to cause structural changes enabling photoconversion. Five other substitutions, S21T, F99Y, L124M, K162R, and P186S, lie far away from the chromophore, presumably affecting its folding and brightness (Figure 3B).

Analogously to PSmOrange, in PSmOrange2, position 64 is occupied by Leu pointing out at the importance of a hydrophobic residue in this position. PSmOrange2 differs from PSmOrange by total six substitutions, only two of which, F65I and A217S, are located close to the chromophore (Figure 3C). Replacement of aromatic F65 (present in both mOrange and PSmOrange) by aliphatic I65 is accompanied by a dramatic decrease of the amount and intensity of light required for an efficient photoconversion. Four other residues are located on the surface of the protein. S217 in PSmOrange2, forms a strong H-bond with catalytic E215 and both, S217 and E215, adopt two conformations (Figure 4C). In conformation “A”, the side chain of S217 is oriented away from E215 and forms a H-bond with the main chain carbonyl of Y72. This makes E215 move away from the chromophore, causes break of a H-bond between Oε2 atom of E215 and N2 atom of the chromophore (4.4 Å), and provides formation of a new H-bond between the side chains of E215 and Q42. In conformation “B”, S217 points towards E215, forming a strong H-bond (2.6 Å) with its side chain and pushing E215 closer to the chromophore, facilitating H-bonding between E215 and nitrogen of imidazolinone ring (3.1 Å) that connects it with the protein matrix. In parental PSmOrange, position 217 is occupied by Ala, as a result, E215 is moved away from the chromophore forming no H-bond with it (E215-Oε2 – TYG-N2 distance is 3.9 Å) (Figure 4C).

Photoconverted far-red forms of PSmOrange and PSmOrange2 are relatively unstable and undergo 50% degradation within several days after photoconversion, presumably due to the overall oxidation and degradation of the protein [20], [21]. This instability made impossible obtaining the suitable crystals of the far-red forms. Photoconversion of the crystals of PSmOrange and PSmOrange2 attempted in crystallization drops with added potassium ferricyanide was also unsuccessful presumably due to a poor diffusion of the oxidant in the crystals.

**Figure 5:**
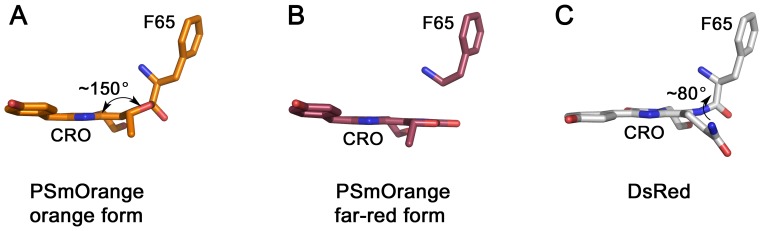
The structures of the PSmOrange and DsRed chromophores. (**A**) The structure of the orange form of the PSmOrange chromophore. (**B**) The modeled structure of the photoconverted far-red form of the PSmOrange chromophore. (**C**) The structure of the DsRed chromophore.

In an earlier work on PSmOrange, it was shown that mass-spectrometry analysis of the photoconverted chromophore-containing peptide was in a good agreement with the mechanism of PSmOrange photoswitching involving a cleavage of the polypeptide chain between the main chain carbonyl and Cα of F65 [20]. It has been suggested that the PSmOrange2 photoconversion is similar and may consist of a break of the polypeptide chain between the main chain carbonyl and Cα of I65. [21]. To get an idea of the structural changes taking place in far-red forms of PSmOrange and PSmOrange2, we have modeled their structures based on the corresponding non-photoconverted proteins (Figure 5). The modeling revealed that the cleavage of Cα-C bond of F65/I65 eliminates the strain imposed on the chromophore by internal α-helix of the protein, causing Cα1 atom of the chromophore to adopt a true planar sp^2^-geometry (Figure 5B). As a result, the oxazole ring of the chromophore moves away from F65/I65 and becomes positioned in–plane with two other rings, significantly improving the efficiency of the overall chromophore conjugation. Oxidation of the hydroxyl of 2-hydroxy-dihydrooxazole to carbonyl puts this group in plane with the rest of the chromophore; it faces (but is not embedded in) the hydrophobic pocket formed by the residues F14, V16, A44, L46, F/I65, and Y120. Appearance of C = O group extends the chromophore conjugation system, providing an additional contribution to the red shift of the fluorescence of the photoconverted PSmOrange and PSmOrange2.

## Discussion

Continuing progress in fluorescence imaging requires probes with additional colors and properties optimized for emerging imaging techniques. A typical process for the development of FPs with desired properties includes rational design followed by random mutagenesis. Rational design relies on the accumulated knowledge about the influence of the certain amino acid residues in the immediate chromophore environment on FP properties. At this stage, first key mutations responsible for the desired target properties of the FP are introduced. This minimally essential variant is then subjected to a directed molecular evolution aimed at optimization of an overall performance of FP.

**Figure 6:**
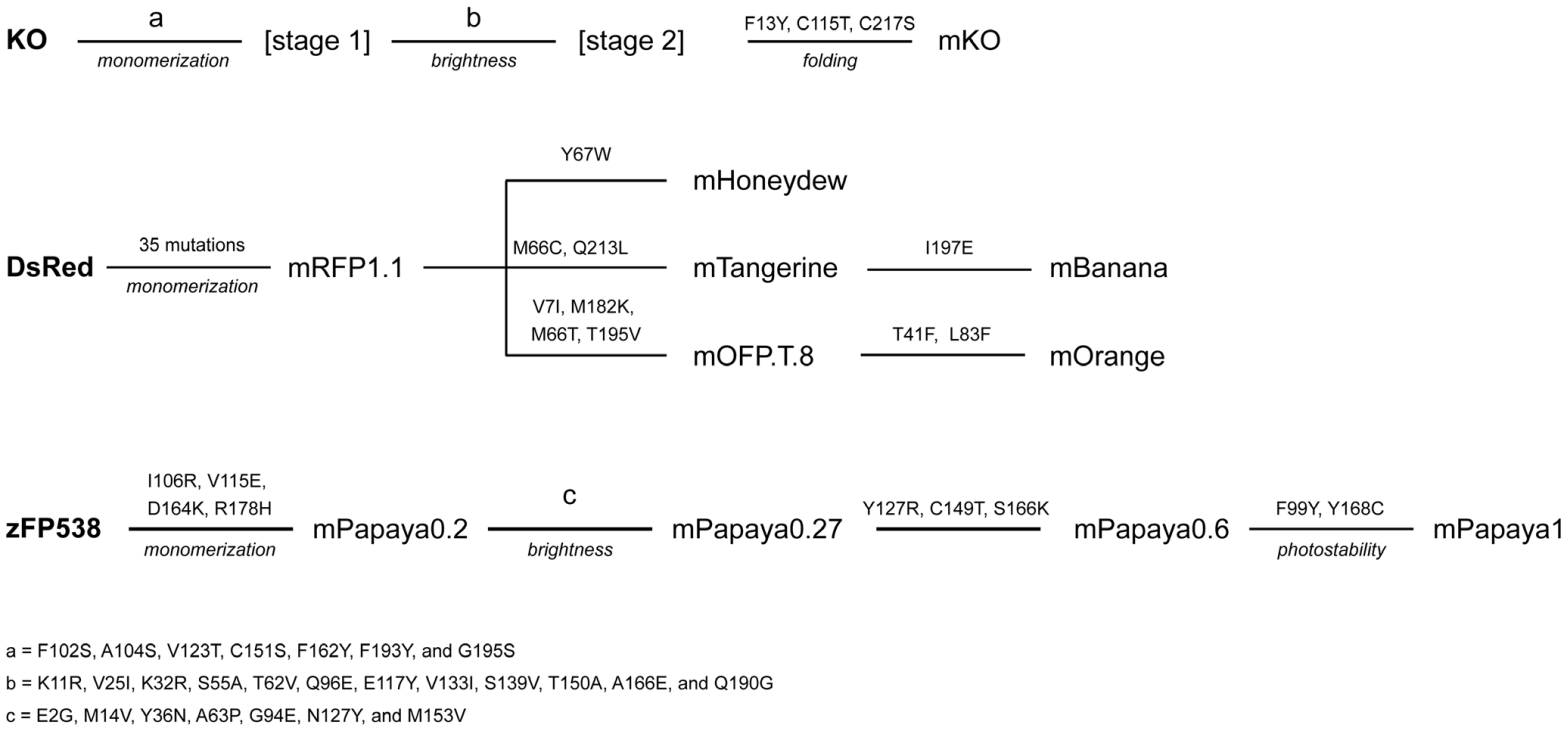
An evolution of orange fluorescent proteins derived from KO, DsRed and zFP538 with mutations critical to the phenotype of each variant.

Development of monomeric variant of KO started from the introduction of seven point mutations on the surface of the protein (F102S, A104S, V123T, C151S, F162Y, F193Y, and G195S) known to disrupt the tetrameric interfaces of DsRed [32]. Here and below the numbering for amino acids is adopted from original publications. Additional mutagenesis that followed found two other sets of mutations, twelve (K11R, V25I, K32R, S55A, T62V, Q96E, E117Y, V133I, S139V, T150A, A166E, and Q190G) and three (F13Y, C115T, and C217S), to improve mKO's brightness and folding efficiency, respectively [4] (Figure 6).

The development of monomeric FPs of different colors collectively named mFruits was also started from the disruption of the interfaces of tetrameric DsRed to yield mRFP1.1 [2] that was further used as a template for the development of monomeric FPs of multiple colors [2]. Figure 6 shows the key residues providing orange emission of FPs derived from DsRed. Y67W is the key substitution converting red mRFP1.1 into orange FP, mHoneydew. Two substitutions, M66C and Q213L, are required to transform mRFP1.1 in mTangerine, which in turn can be converted into mBanana by additional I197E mutation. Four point mutations, V7I, M182K, M66T, and T195V, are required to convert mRFP1.1 in the OFP prototype, mOFP.T.8. Two additional mutations, T41F and L83F, converted mOFP.T.8 in a highly acclaimed bright OFP mOrange. In all cases, rational design has been followed by directed evolution approach to perfect newly generated FPs - enhance their brightness, folding efficiency, and photostability.

The other vivid example of rational design and directed evolution approach combination is mPapaya1 [10] (Figure 6). First, to design monomeric version of zFP538, four mutations (I106R, V115E, D164K, and R178H) were introduced at the interfaces between its subunits (mPapaya0.2). Then, to restore the brightness, lost during monomerization, the protein was subjected to four rounds of directed evolution that introduced seven additional mutations (mPapaya0.27). To further improve monomeric character of mPapaya0.27, additional three mutations, Y127R, C149T, and S166K were made resulting in truly monomeric mPapaya0.6. Finally, to improve photostability mPapaya0.6 was subjected to additional cycle of directed evolution resulting in mPapaya0.6/F99Y/Y168C designated as mPapaya1.

Bright monomeric FPs serve as templates for the development of FPs with special properties, such as FPs with a large Stokes shift, photoswitching FPs and Fluorescent timers [1]. Currently available LSSFPs include green T-Sapphire [33], yellow mAmetrine [34], orange LSSmOrange [19], and red LSSmKate1 [35], LSSmKate2 [35], and mKeima1 [36]. Engineering of LSSFPs aims at providing an ESPT between Tyr hydroxyl of the chromophore and its nearest environment. The common mechanism implies that the chromophore is initially protonated. Upon excitation, the proton is transferred to a nearby proton acceptor either directly or with the help of an H-bond network. The anionic chromophore intermediate emits a photon, returns to the ground state, and becomes protonated.

In wild-type GFP, two ESPT pathways have been reported: the major one follows a well-defined chromophore-Water22-S205-E222 proton wire [37], while a less efficient one is based on a change of T203 conformation promoting proton ejection out of the β-barrel [38]. In the ground state, the chromophore is reprotonated by E222 acquiring a proton from the outside by the E5 entry pathway, located near the N-terminus of GFP [38]. In green T-Sapphire and yellow mAmetrine, ESPT follows the same pathway as the major one of wtGFP. In LSSmKate1, ESPT is realized as the proton shuttle between the *cis*-chromophore and E160, whereas in LSSmKate2, ESPT pathway comprises *trans*-chromophore, S158, and D160 [39]. Similarly, in mKeima, proton is transferred along the proton wire *cis*-chromophore-S146-D161 [40], [41]. All elements of the mKeima proton wire are present in LSSmOrange (*cis*-chromophore) indicating to a similar mechanism of ESPT; this suggestion has been confirmed by site-directed mutagenesis. Earlier, we have demonstrated that both LSSmOrange/S146A and LSSmOrange/D161A mutants disturb LSS phenotype and LSSmOrange/D161A displays a regular Stokes shift (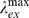/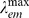 550/565 nm) similar to that of mOrange [19]. Parental mOrange has position 146 already occupied by Ser, which is one of two essential residues required for ESPT.

PSFPs are widely used for tracking intracellular proteins, organelles, and individual cells [42], [43]. Fluorescence of PSFPs is switched from one color to another by the light of a specific wavelength. A majority of PSFPs including Dendra2 [44], mEos2 [45], Kaede [46], mKikGR [47], mClavGR2 [48] and their derivatives change their fluorescence from green to red after irradiation with a relatively phototoxic violet light (390−410 nm). The blue-to-green proteins photoswitchable with this light, PSCFP and PSCFP2, are also available [44]. Photoconversion of PSmOrange and PSmOrange2 with 480–540 nm light makes these proteins the first PSFPs efficiently photoswitched with non-phototoxic visible light [20], [21]. SDS-PAGE and mass spectrometry results demonstrated that PSmOrange photoconversion is accompanied by a cleavage of polypeptide chain between the chromophore and F65 and by oxidation of the hydroxyl of 2-hydroxy-dihydrooxazole ring of the chromophore to carbonyl [20]. This modification strongly affects the conjugation system of the chromophore - it enables the chromophore to recover its distorted planarity around Cα1 atom and adds C = O bond to the conjugation resulting in a substantial (∼90 nm) bathochromic shift of photoconverted PSmOrange absorbance/emission bands (Figure 5B). This is not the case for the other RFPs, such as DsRed (*Discosoma sp*.) (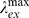/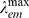 558/583 nm) [5], eqFP578 (*Entacmaea quadricolor*) (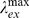/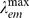 552/578 nm) [49], or zFP574 (*Zoanthus sp*.) (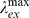/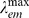 553/574 nm) [50]: in all these RFPs, carbonyl of the amino acid preceding the chromophore is not coplanar with it (Figure 5C). In fact, for these proteins a ∼80° dihedral angle between the carbonyl group and the chromophore makes the conjugation extremely inefficient. This carbonyl cannot be coplanar with the chromophore due to the bent in the central α-helix embedding the chromophore. Inefficient conjugation results in a blue-shift of fluorescence of DsRed, eqFP578, and zFP574 relative to that of photoswitched PSmOrange.

It has been demonstrated that position 64 mutation is minimally required for appearance of photoswitchable properties [20]. In mOrange, Q64 forms an H-bond with R95; this H-bond is absent in PSmOrange, where Q64 is replaced with hydrophobic Leu (Figure 4B), suggesting that the absence of H-bond between residues 64 and 95 facilitates orange to far-red photoconversion of PSmOrange chromophore.

Monomeric PSmOrange2 has been developed as an improved version of PSmOrange with faster and more efficient photoswitching. Its photoconversion could be achieved with common two-photon lasers and it is, therefore, much more user-friendly than parental PSmOrange. Moreover, PSmOrange2 could be used as an acceptor in Förster resonance energy transfer with green fluorescent donors. This fact together with its high efficiency of photoconversion and fast photoswitching kinetics enabled photoswitching of PSmOrange2 via FRET from the donor FP. The brightness of orange and far-red forms of PSmOrange2 is 1.9-fold and 1.2-fold lower than that of respective PSmOrange forms (Table 1). However, its photoswitching contrast, which is 9-fold higher than that of PSmOrange, a substantially higher efficiency of PSmOrange2 photoconversion than that of the parental protein makes PSmOrange2 a better tag. The key mutation that provided dramatic improvement of PSmOrange2 photoconversion is F65I. Replacement of aromatic F65 with aliphatic I65 and decrease of the light energy required for photoswitching imply that I65 facilitates backbone cleavage and decreases the activation barrier of photoconversion. Modeling of photoconverted PSmOrange structure revealed that the residue 65 and the oxidized chromophore move away from each other after the cleavage of Cα1-C bond (Figure 5B). A lower energy required for a photoconversion of PSmOrange2 suggests that less bulky side chain of I65 enables an easier stabilization of detached Ile within its immediate environment than of a bulkier and more rigid F65 in PSmOrange. Substitution A217S increases quantum yield of the orange form of PSmOrange2 [21]. This is presumably caused by an increased charge separation in the chromophore, taking place as a result of a new H-bond between E215 and its N2 atom. Formation of this H-bond occurs only in the presence of S217 that makes E215 move closer to the chromophore. A similar H-bond between N2 and E215 has been described earlier for rsTagRFP where it enabled formation of a fluorescent zwitter-ionic species [51].

## Conclusions

Here we presented the structural analysis of three recently developed OFPs with novel phenotypes such as a large Stokes shift and photoswitchability. Spectroscopic characteristics provided the insights in the properties of these OFPs and extensive mutagenesis identified the residues responsible for the photochemical changes. Crystallographic structures presented here revealed how the ensemble of key mutations introduced in the parental FPs enabled those changes.

For LSSmOrange, ESPT occurs over the *cis*-chromophore-S146-D161 pathway and is caused by three key substitutions: I161D, M163L, and W143M. The W143M replacement provided a shift of the side chain of L165 towards protein interior, and both W143M and M163L improve the chromophore planarity. Rotation of the chromophore around χ1 angle, arising from these two mutations, causes the change of S146 side chain conformation. As a result, S146 acts as a mediator for ESPT from *p-*hydroxyphenyl ring of the chromophore to carboxyl group of D161.

For PSmOrange and PSmOrange2, Q64L mutation is minimally required for appearance of photoswitchable properties. The structural data demonstrated that this mutation disrupts an H-bond between positions 64 and R95 facilitating orange to far-red photoconversion of PSmOrange chromophore. The photoconversion itself is accompanied by cleavage of Cα1-C bond of F/I65, resulting in recovery of the distorted chromophore planarity, and by oxidation of OH-group of 2-hydroxy-dihydrooxazole to C = O, resulting in the extension of the chromophore conjugation system. This was the primary source of a substantial (∼90 nm) bathochromic red shift of fluorescence in photoconverted PSmOrange and PSmOrange2. Substitution A217S in PSmOrange2 induces the shift of E215 closer to the chromophore and cause formation of its H-bond with N2 atom, increasing polarization of the chromophore and its quantum yield.

**Figure 7:**
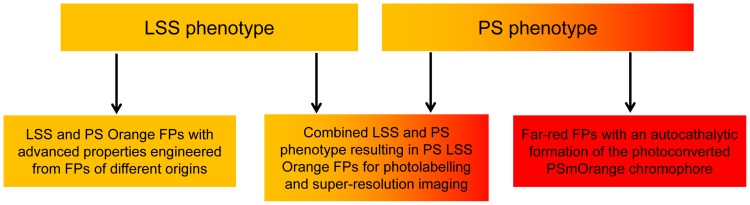
Possibilities for the future design of advanced orange and far-red fluorescent proteins.

The detailed analysis of the chromophore structures and chromophore transformations in LSSmOrange and PSmOrange/PSmOrange2 will serve as a basis for engineering of future advanced FPs (Figure 7). First, enhanced OFP variants could be designed by introducing ESPT or photoconversion pathways in OFPs of different origins including wild-type OFPs. The key positions for mutations (Figure S1) can be used to introduce substitutions in new protein templates at the first step, followed by random mutagenesis. Second, novel OFP phenotypes can be obtained by combining different pathways of OFP chromophore transformations in a single FP. We anticipate that the LSS phenotype can be combined with PS phenotypes to engineer PS LSS OFP. This future FP should further advance the multicolor photolabelling in wide-field fluorescence microscopy and in super-resolution imaging of live cells. Lastly, another engineering goal could be a development of a FP with the autocatalytic formation of the photoconverted far-red PSmOrange chromophore. Such permanently fluorescent far-red FP is highly desirable as a genetically-encoded probe for non-invasive deep-tissue *in vivo* imaging.

The coordinates and structure factors were deposited in the Protein Data Bank under the accession codes 4Q7R, 4Q7T and 4Q7U for LSSmOrange, PSmOrange and PSmOrange2, respectively.

## Supporting Information

Figure S1 **Amino acid alignment of orange fluorescent proteins.** The chromophore-forming tri-peptides are highlighted in yellow.
(DOC)
