# Supplementary material for: Orange Fluorescent Proteins: Structural Studies of LSSmOrange, PSmOrange and PSmOrange2
Source: PLoS One. 2014 Jun 24;9(6):e99136. doi: 10.1371/journal.pone.0099136 (PMC4068994; doi:10.1371/journal.pone.0099136)
Supplement: Figure S1 — Amino acid alignment of orange fluorescent proteins. The chromophore-forming tri-peptides are highlighted in yellow. (DOC) [file pone.0099136.s001.doc]

**Orange fluorescent proteins: structural studies of LSSmOrange, PSmOrange and PSmOrange2**

Sergei Pletnev, Daria M. Shcherbakova, Oksana M. Subach, Nadya V. Pletneva, Vladimir N. Malashkevich, Steven C. Almo, Zbigniew Dauter and Vladislav V. Verkhusha

SUPPLEMENTARY INFORMATION

10 20 30 40 50 60

| | | | | |

zFP538 -----MAHSKHGLKEEMTMKYHMEGCVNGHKFVITGEGIGYPFKGKQTINLCV--IEGGP

mPapaya1 MVSKGEGQSKHGLKEEMTVKYHMEGCVNGHKFVITGEGIGNPFKGKQTANLCV--IEGGP

phiYFP -----MSSGALLFHGKIPYVVEMEGNVDGHTFSIRGKGYGDASVGKVDAQFICT---TGD

phiYFPv -----GSSGALLFHGKIPYVVEMEGNVDGHTFSIRGKGYGDASVGKVDAQFICT---TGD

TagYFP ----MVSKGEELFAGIVPVLIELDGDVHGHKFSVRGEGEGDADYGKLEIKFICT---TGK

KO ---------MSVIKPEMKMKYFMDGSVNGHEFTVEGEGTGKPYEGHQEMTLRVTMAKGGP

mKO --------MVSVIKPEMKMRYYMDGSVNGHEFTIEGEGTGRPYEGHQEMTLRVTMAKGGP

mKO2 --------MVSVIKPEMKMRYYMDGSVNGHEFTIEGEGTGRPYEGHQEMTLRVTMAEGGP

mKOk ---------MSVIKPEMKMRYYMDGSVNGHEFTIEGEGTGRPYEGHQEMTLRVTMAEGGP

mBanana MVSKGEENNMAVIKEFMRFKVRMEGSVNGHEFEIEGEGEGRPYEGTQTAKLKVT--KGGP

E2_Orange --MDSTEN---VIKPFMRFKVHMEGSVNGHEFEIEGEGEGKPYEGTQTAKLQVT--KGGP

mHoneydew MVSKGEE----VIKEFMRFKVRMEGSVNGHEFEIEGEGEGRPYEGTQTAKLKVT--KGGP

mOrange MVSKGEENNMAIIKEFMRFKVRMEGSVNGHEFEIEGEGEGRPYEGFQTAKLKVT--KGGP

mOrange2 MVSKGEENNMAIIKEFMRFKVRMEGSVNGHEFEIEGEGEGRPYEGFQTAKLKVT--KGGP

LSSmOrange MVSKGEENNMAIIKEFMRFKVRMEGSVNGHEFEIEGEGEGRPYEGFQTVKLKVT--KGGP

PSmOrange MVSKGEENNMAIIKEFMRFKVRMEGTVNGHEFEIEGEGEGRPYEGFQTAKLKVT--KGGP

PSmOrange2 MVSKGEENNMAIIKEFMRFKVHMEGTVNGHEFEIEGEGEGHPYEGFQTAKLKVT--KGGP

70 80 90 100 110 120

| | | | | |

zFP538 LPFSEDILSAGFKYGDRIFTEYPQ--DIVDYFKNSCPAGYTWGRSFLFEDGAVCICNVDI

mPapaya1 LPFSEDILSPGFKYGDRIFTEYPQ--DIVDYFKNSCPAGYTWERSYLFEDGAVCRCNVDI

phiYFP VPVPWSTLVTTLTYGAQCFAKYGP--ELKDFYKSCMPEGYVQERTITFEGDGV--FKTRA

phiYFPv VPVPWSTLVTTLTYGAQCFAKYGP--ELKDFYKSCMPDGYVQERTITFEGDGN--FKTRA

TagYFP LPVPWPTLVTTLTYGVQCFARYPKHMKMNDFFKSAMPEGYIQERTILFQDDGK--YKTRG

KO MPFSFDLVSHTFCYGHRPFTKYPE--EIPDYFKQAFPEGLSWERSLQFEDGGF--AAVSA

mKO MPFAFDLVSHVFCYGHRPFTKYPE--EIPDYFKQAFPEGLSWERSLEFEDGGS--ASVSA

mKO2 MPFAFDLVSHVFCYGHRVFTKYPE--EIPDYFKQAFPEGLSWERSLEFEDGGS--ASVSA

mKOk MPFAFDLVSHVFCYGHRVFTKYPE--EIPDYFKQAFPEGLSWERSLEFEDGGS--ASVSA

mBanana LPFAWDILSPQFCYGSKAYVKHPT--GIPDYFKLSFPEGFKWERVMNFEDGGV--VTVAQ

E2_Orange LPFAWDILSPQFTYGSKAYVKHPA--DIPDYKKLSFPEGFKWERVMNFEDGGV--VTVTQ

mHoneydew LPFAWDILSPQFMWGSKAYVKHPA--DIPDYLKLSFPEGFKWERVMNFEDGGV--VTVTQ

mOrange LPFAWDILSPQFTYGSKAYVKHPA--DIPDYFKLSFPEGFKWERVMNFEDGGV--VTVTQ

mOrange2 LPFAWDILSPHFTYGSKAYVKHPA--DIPDYFKLSFPEGFKWERVMNYEDGGV--VTVTQ

LSSmOrange LPFAWDILSPQFTYGSKAYVKHPA--DIPDYLKLSFPEGFKWERVMNFEDGGV--VTVTQ

PSmOrange LPFAWDILSPLFTYGSKAYVKHPA--DIPDYFKLSFPEGFKWERVMNYEDGGV--VTVTQ

PSmOrange2 LPFAWDILSPLITYGSKAYVKHPA--DIPDYFKLSFPEGFKWERVMNYEDGGV--VTVTQ

130 140 150 160 170 180

| | | | | |

zFP538 TVSVKENCIYHKSIFNGMNFPADGPVMKK-MTTNWEASCEKIMPVPKQGILKGDVSMYLL

mPapaya1 TVSEKENCIYHKSIFRGVNFPADGPVMKK-MTTNWEASTEKIVPVPKQGILKGKVKMCLL

phiYFP EVTFENGSVYNRVKLNGQGFKKDGHVLGKNLEFNFTPHCLYIWGDQANHGLKSAFKIMHE

phiYFPv EVTFENGSVYNRVKLNGQGFKKDGHVLGKNLEFNFTPHCLYIWGDQANHGLKSAFKICHE

TagYFP EVKFEGDTLVNRIELKGKDFKEDGNILGHKLEYSFNSHNVYITPDKANNGLEVNFKTRHN

KO HISLRGNCFEHKSKFVGVNFPADGPVMQN-QSSDWEPSTEKITTC--DGVLKGDVTMFLK

mKO HISLRGNTFYHKSKFTGVNFPADGPIMQN-QSVDWEPSTEKITAS--DGVLKGDVTMYLK

mKO2 HISLRGNTFYHKSKFTGVNFPADGPIMQN-QSVDWEPSTEKITAS--DGVLKGDVTMYLK

mKOk HISLRGNTFYHKSKFTGVNFPADGPIMQN-QSVDWEPSTEKITAS--DGVLKGDVTMYLK

mBanana DSSLQDGEFIYKVKLRGTNFPSDGPVMQK-KTMGWEASSERMYPE--DGALKGEIKMRLK

E2_Orange DSSLQDGTFIYHVKFIGVNFPSDGPVMQK-KTLGWEPSTERLYPR--DGVLKGEIHKALK

mHoneydew DSSLQDGEFIYKVKLRGTNFPSDGPVMQK-KTMGWAATTERMYPE--DGALKGEIKMRLK

mOrange DSSLQDGEFIYKVKLRGTNFPSDGPVMQK-KTMGWEASSERMYPE--DGALKGEIKMRLK

mOrange2 DSSLQDGEFIYKVKLRGTNFPSDGPVMQK-KTMGWEASSERMYPE--DGALKGKIKMRLK

LSSmOrange DSSLQDGEFIYKVKLRGTNFPSDGPVMQK-KTMGMEASSERMYPE--DGALKGEDKLRLK

PSmOrange DSSLQDGEFIYKVKMRGTNFPSDGPVMQK-KTMGWEASSERMYPE--DGALKGEIRMRLK

PSmOrange2 DSSLQDGEFIYKVKMRGTNFPSDGPVMQK-KTMGWEASSERMYPE--DGALKGEIRMRLK

190 200 210 220 230 240

| | | | | |

zFP538 LKD--GGRYRCQFDTVYKAKSV-PSKMPEWHFIQHKLLREDRSDAKNQKWQLTEHAIAFP

mPapaya1 LKD--GGRYHCQFDTVYKAKSV-PSKMPEWHFIQHKLLREDRSDAKNQKWQLTEHAIAGM

phiYFP ITGSKEDFIVADHTQMNTPIGGGPVHVPEYHHITYHVTLSKDVTDHRDNMSLVETVRAVD

phiYFPv ITGSKGDFIVADHTQMNTPIGGGPVHVPEYHHMSYHVKLSKDVTDHRDNMSLKETVRAVD

TagYFP IEG--GGVQLADHYQTNVPLGDGPVLIPINHYLSYQTDISKDRNEARDHMVLLESVSA--

KO LAG--GGNHKCQFKTTYKAAKK-ILKMPQSHFIGHRLVRKTEGNIT----ELVEDAVAHC

mKO LEG--GGNHKCQFKTTYKAAKK-ILKMPGSHYISHRLVRKTEGNIT----ELVEDAVAHS

mKO2 LEG--GGNHKCQMKTTYKAAKE-ILEMPGDHYIGHRLVRKTEGNIT----EQVEDAVAHS

mKOk LEG--GGNHKCQFKTTYKAAKE-ILEMPGDHYIGHRLVRKTEGNIT----EQVEDAVAHS

mBanana LKD--GGHYSAETKTTYKAKK--PVQLPGAYIAGEKIDITSHNEDYT-IVELYERAEGRH

E2_Orange LKG--GGHYLVEFKTIYMAKK--PVKLPGYYYVDSKLDITSHNEDYT-VVEQYERAEARH

mHoneydew LKD--GGHYDAEVKTTYMAKK--PVQLPGAYKIDGKLDITSHNEDYT-IVEQYERAEGGH

mOrange LKD--GGHYTSEVKTTYKAKK--PVQLPGAYIVGIKLDITSHNEDYT-IVEQYERAEGRH

mOrange2 LKD--GGHYTSEVKTTYKAKK--PVQLPGAYIVDIKLDITSHNEDYT-IVEQYERAEGRH

LSSmOrange LKD--GGHYTSEVKTTYKAKK--PVQLPGAYIVDIKLDITSHNEDYT-IVEQYERAEGRH

PSmOrange LKD--GGHYTSEVKTTYKAKK--SVQLPGAYIVGIKLDITSHNEDYT-IVEQYERAEGRH

PSmOrange2 LKD--GGHYTSEVKTTYKAKK--SVLLPGAYIVGIKLDITSHNEDYT-IVEQYERSEARH

250 260

| |

zFP538 S-----ALA-------------

mPapaya1 D-----ELYK------------

phiYFP ----CRKTYL------------

phiYFPv ----CRKTYDFDAGSGDTSLIS

TagYFP ----CSHTHGMDELYR------

KO ----------------------

mKO ----------------------

mKO2 ----------------------

mKOk ----------------------

mBanana STGGMDELYK------------

E2_Orange ------HLFQ------------

mHoneydew STGGMDELYK------------

mOrange STGGMDELYK------------

mOrange2 STGGMDELYK------------

LSSmOrange STGGMDELYK------------

PSmOrange STGGMDELYK------------

PSmOrange2 STGGMDELYK------------

**Figure S1. Amino acid alignment of orange fluorescent proteins.** The chromophore-forming tri-peptides are highlighted in yellow.
